# Fever and the Use of Antipyretics

**Published:** 1888-11

**Authors:** F. W. McRae

**Affiliations:** Atlanta, Ga., Lecturer on Diseases of Children and Demonstrator of Anatomy in the Atlanta Medical College


VOL. V. NOVEMBER, 1888. NO. 9.

### Original Communications.

### FEVER AND THE USE OF ANTIPYRETICS.

BY F. W. M'RAE, M. D., ATLANTA, GA.,

Lecturer on Diseases of Children and Demonstrator of Anatomy in the Atlanta Medical College.

Ever since Leibermeister's able advocacy of the antipyretic treatment of fever, it has been the general practice with the profession to give antipyretics of one sort or another in almost all diseases where the temperature remained at or about 103° F. for any length of time. Leibermeister believed and taught that the various pathological changes of the glandular and muscular systems resulting in fatty degenerations—found on examination of bodies of those who had died with typhoid and other infectious diseases—were the direct effects of excessive temperature. Since then his views have been accepted by the great mass of the profession, who have directed their treatment accordingly. It has been, and is still, claimed by a great many authors and clinicians that the mortality in typhoid fever may be reduced to almost in by keeping the temperature within certain limits either by the use of the cold bath or other antipyretic agents. Leibermeister, Senator and other experimenters have obtained experimental results conclusively demonstrating to their minds the correctness of this theory.

Clinical experience, however, has furnished abundant proof of the impotence of antipyretics in not a small proportion of cases. The fact that many fatal cases of typhoid fever presenting the characteristic visceral lesions have uniformly low temperatures throughout their entire course has been ignored by most of those authors who consider the hyperpyrexia the chief source of danger and indication for treatment.

There have been, however, all along quite a number of eminent medical men who have dissented from the teachings of Leibermeister and his followers—some even considering the increased temperature a conservative process.

The Cartwright lectures, delivered recently at the College of Physicians and Surgeons by Wm. H. Welch, M. D., Professor of Pathology in John Hopkins' University, have thrown a great deal of light on this subject. He demonstrated the fact that animals can resist for a longer time a much higher temperature than has heretofore been considered compatible with life. The animals used in these experiments were rabbits. One rabbit, kept with an average rectal temperature of 107.3° F. for three weeks, then taken from the box used in the experiment, remained perfectly well for ten days, was used in another experiment. Another, kept with an average temperature of 106.6° F. for the same length of time, then killed, "presented well-marked fatty degeneration of the heart, liver and kidneys."

Welch showed by still further observation under the microscope of the muscular fibers taken from the rabbit's heart that the heart muscle is capable of rythmical and normal contractions even after there is well-marked evidence of fatty degeneration.

It is now conceded by most of those who have given especial thought and attention to the subject that the temperature is under the direct control of the thermal centers, though the question is still *subjudice*—the thermo-genic, thermo-inhibitor and thermolytic centers and nerves, by concert of action, keeping the bodily heat at the normal standard, notwithstanding the constant change of environment. In fever this nervous coördination is disturbed. Wood has shown that while more heat is generated by an animal with fever than would be produced under like conditions of diet and rest, there is not more heat produced than would be by the same animal in health on a full diet.

There is no positive evidence that the digestive disorders accompanying typhoid and other fevers are the result of increased temperature, but rather of infection. The nervous symptoms accompanying these diseases must also be referred to the same cause.

Many cases are on record of patients who have withstood excessive temperatures for several days or even weeks without marked ill effects. (Dr. Teal's case, 108°-122° for nine weeks.) In relapsing fever also we have evidence of the resistance of the system to continuous high temperature.

It seems to me—and I think the position well sustained by experimental medicine and clinical experience—that the pathological visceral changes are due in much greater degree to infection than to increased temperature, though there is no doubt but that the combined influence of the two is much greater than either alone.

Whichever view of the subject we may take, we know that in the continued fevers, especially in typhoid, we have to deal with grave pathological changes which seriously affect the issue. We know that whatever the cause of the fever there is increased chemical activity of the constituent elements of the body and augmented muscular friction produced by the pyrogenic substances.

Perhaps the first organ affected is the liver. It is one of the first organs whose functional activity is interfered with, thus producing biliousness and impaired digestion. Early in these fevers it presents palpable cellular changes soon terminating in true and extensive parenchymatous degeneration. Recent physiological investigations have shown conclusively that the liver stands guard over the portal circulation to prevent the entrance into the general circulation of deleterious or dangerous substances from the alimentary canal.

Thus we can readily understand how the irritating products of imperfect digestion, and effete material from the alimentary canal entering the general circulation through the inefficiency of the functionally or organically diseased liver, increase the chemico-physiological processes, and consequently the heat production. This effete matter must be eliminated from the body chiefly by the skin and kidneys. We can readily see how inactivity of either or both would jeopardize the integrity of the heart, lungs, spleen and other viscera, and ultimately every tissue of the organism. The poison acting through the blood upon the glandular organs causing functional or organic derangement may be, and most probably is, the cause rather than the effect of increased temperature.

Heat is eliminated from the system by the skin and lungs, about (80) eighty per cent. by the former. Now it seems to me that conservative antipyretic treatment should have two objects: first, to diminish heat production; second, to increase heat dissipation. Now do the so-called antipyretics accomplish both or either of these results? If so do they do it safely? I do not believe they do in the large proportion of cases.

Experimenters have established the fact that as a class the antipyretics, such as antifebrine, antipyrine, thallin, etc., produce marked intersticial changes in the kidneys, if not in other organs, in full physiological doses. The urine is diminished in quantity; its specific gravity and the urates are increased; it presents a smoky appearance and frequently contains casts.

Most observers are agreed also in attributing to them the power of producing marked blood changes. The red blood corpuscles are diminished in number and the oxyhæmoglobin is converted into methæmoglobin.

Porter, of New York, in a paper read before the "Ontario Medical Association," advances the theory that by causing a retention of excrementitious substances the chemico-physiological processes are augmented and the temperature is ultimately increased instead of diminished by their use. He also asserts that deaths with excessively high temperatures are much more frequent now than before the introduction of this class of remedies. It seems rational to believe that agents which act so powerfully on healthy tissues would exert a much greater influence on tissues already rendered vulnerable by disease. If these facts were not borne out in part at least by clinical experience, there would be good grounds for disregarding the teachings of experimental medicine. Their use has not diminished the death rate in typhoid fever nor has it shortened the duration of the disease. The cold bath, too, I think, is in many cases powerful for evil as well as for good. While many patients suffer no inconvenience from its application, there are not a few who are injured by it. I think there are five well-grounded contra-indications:

1st. The shock which it produces.

2nd. We know that the application of cold to the surface increases the production of heat.

3rd. It causes contraction of the cutaneous capillaries, and thus diminishes natural heat dissipation and the elimination of effete material by the skin.

4th. It causes passive congestion of the viscera and predisposes to stasis and to hæmorrhage.

5th. The wear and tear of the patient when it is so essential to harbor every particle of vitality.

My observation has been as soon as the baths have been discontinued the temperature soon reaches or exceeds its former height.

These objections do not apply, of course, to the short, rapid elevations of temperature such as we have in insolation. One of the prime objections to the use of antipyretics and the cold bath is the increased danger of heart failure, the thing, above all others, which we wish to obviate.

I believe the indications are best fulfilled by the use of diuretics, diaphoretics, agents which prevent intestinal fermentation, and alcohol.

I believe diaphoretics are antipyretics, and safe ones, because they accomplish the results which nature is endeavoring to bring about. They increase heat dissipation, nature's method of preserving heat equilibrium, and cutaneous elimination.

Refrigerant diuretics are also useful; they act as antipyretics by eliminating from the system the products of nitrogenous metabeolism and imperfect oxidation and keep the kidneys in a healthy condition.

By the use of antizymotics internally we can, to a considerable degree, prevent intestinal fermentation, and thus prevent this class of pyrogenic substances from gaining entrance into the blood. But by far the most important of internal remedies is alcohol. It stimulates digestion and is rapidly absorbed from the stomach. It is taken up by the tissues and presents itself a vicarious offering for oxidation in lieu of the tissues themselves, and in its oxidation water is evolved. It stimulates and strengthens the heart's action, thus tending to prevent heart failure; and lastly, it dilates the cutaneous capillaries, thus reducing fever by radiation.

The tepid bath is also an invaluable adjunct. It softens and cleanses the skin, increases sensible and insensible perspiration, allays nervous irritability and relieves visceral congestion. In conclusion I will quote the closing paragraph of Welch's lectures, the force of which we must all admit:

"I cannot conclude this course of lectures without saying a word on a subject which must engage the attention of every one who gives thought to the nature of fever. What is the significance of fever is a question which thrusts itself upon us no less than it has upon physicians in all ages.

"Unfortunately we cannot to-day, any more than could our predecessors, give other than a speculative answer to this question. There have been in all ages enlightened physicians who have held the opinion that fever is a process which aids in the elimination or destruction of injurious substances which gain access to the body. Under the influence of ideas which sought in increased temperature the origin of the grave symptoms of fever, we have, in recent times, in great part lost sight of the doctrine once prevalent, that there may be in fever a conservative element· There is much which speaks in favor of this doctrine. The real enemy in most fevers is the noxious substance which invades the body, and there is nothing to prevent us from believing that fever is a weapon employed by nature to combat the assault of this enemy. The doctrine of evolution indicates that a process which characterizes the reaction of all warm-blooded animals against the invasion of a host of harmful substances has not been developed to such an extent, and is not retained with such pertinacity without subserving some useful purpose. It is impossible with our present knowledge to say in exactly what way fever accomplishes a useful purpose. There are facts which suggest that in some cases of fever the increased temperature as such may impair the vitality or check the virulence of pathogenic micro-organisms, but there are many circumstances which make it difficult to suppose that this is the agency by which fever usually exerts a favorable action. The supposition seems to me more probable that the increased oxidation of fever aids in the destruction of injurious substances. According to this view the fever-producing agents light the fire which consumes them. It is not incompatible with this conception of fever to suppose that the fire may prove injurious also to the patient and may require the controlling hand of a physician. Time will not permit me to elaborate further the ideas here suggested.

"In the course of these lectures facts have been presented, and others might be drawn from clinical and experimental observations, which favor the hypothesis that fever is, in a certain sense, a conservative process. Unproven and intangible as the hypothesis may seem to some, no apology is needed for bringing to your attention a conception of fever in favor of which much can be adduced and which, if true, is of fundamental importance, both theoretically and practically."

